# Excitation-light blocking filter for photoimmunotherapy tailored to the ORBEYE 4K 3D exoscope

**DOI:** 10.1007/s10147-026-03131-x

**Published:** 2026-07-22

**Authors:** Ryuhei Okada, Hideki Kanebako, Ryosuke Takahashi, Taisei Nagahisa, Shigeru Kondo, Takaaki Sato, Tomoaki Asamori, Kazuchika Ohno, Michiko Machida, Takahiro Asakage

**Affiliations:** 1 Department of Head and Neck Surgery, Institute of Science Tokyo, 1-5-45, Yushima, Bunkyo, Tokyo 113-8519 Japan; 2 Rakuten Medical K.K., Tokyo, Japan

**Keywords:** Photoimmunotherapy, Alluminox, IR700, Photodynamic therapy, Head and neck cancer

## Abstract

**Background:**

Photoimmunotherapy, which involves combining targeted antibodies with light-activatable dyes such as IRDye700DX (IR700), is an emerging cancer treatment modality. Cetuximab-based photoimmunotherapy is approved for the treatment of unresectable head and neck cancer and has been covered by the national health insurance in Japan since 2021. Use of conventional ceiling surgical lighting during photoimmunotherapy is problematic due to concerns about potential cytotoxic effects.

**Methods:**

To ensure safe surgical application, we developed a custom short-pass filter for the ORBEYE surgical exoscope to block IR700 excitation light. The filter’s impact on the color tone was evaluated using human skin and a color checker, while that on the cytotoxicity was assessed in vitro using cetuximab-IR700 and an HSC-3 cell line. The clinical feasibility of using this filter was tested in six patients who underwent short surgical procedures (e.g., tracheostomy, arterial ligation) during photoimmunotherapy.

**Results:**

Recalibrating the white balance after filter placement preserved the natural color tones. In vitro, the filter neutralized the significant cytotoxicity observed under the ORBEYE light. Clinically, the filter could be used safely in all cases with no evidence of postoperative skin damage.

**Conclusion:**

This filter system enhances the safety and feasibility of surgical procedures during photoimmunotherapy.

**Supplementary Information:**

The online version contains supplementary material available at https://doi.org/10.1007/s10147-026-03131-x.

## Introduction

Photoimmunotherapy is a molecular-targeted therapy that utilizes an antibody-photoabsorber conjugate (APC) and an excitation light [1–3]. The photoabsorber used in photoimmunotherapy is IRDye700DX (IR700), which is activated with 690-nm wavelength. The treatment based on this technology is called the Alluminox™ platform. The APC is administered to the patient by intravenous injection, followed subsequently by application of the excitation light to the tumor. The first APC that has been applied in the clinical setting is cetuximab sarotalocan sodium (Akalux®, Rakuten Medical K.K.), which is a cetuximab (anti-epidermal growth factor receptor (EGFR) antibody)-IR700 conjugate (cet-IR700). The administered APC is activated by a laser system (BioBlade®, Rakuten Medical K.K.) under general anesthesia on the following day. Based on favorable results of a phase I/IIa clinical trial conducted in the USA [4] and phase I clinical trial conducted in Japan [5], cetuximab-based photoimmunotherapy for head and neck cancer (HNC) began to be covered by the national health insurance program in Japan in 2021. In the phase IIa part of the above clinical trial, the overall response rate in the 30 participant patients was up to 43.3% [4]. Local edema was reported as one of the most frequent adverse events (AEs). Because laryngeal edema could be fatal, tracheostomy is often performed during photoimmunotherapy. Severe tumor or arterial hemorrhage occurred in 2 (6.7%) patients. Since photoimmunotherapy causes rapid tumor shrinkage, tumor invading the carotid artery should be excluded from receiving this treatment. In case of a possibility of bleeding from the tumor after the treatment, vascular ligation is often performed just prior to the light illumination while the patient is under general anesthesia. As AEs specific to photoimmunotherapy, erythema and rash occurred in 6 (20.0%) and 5 (16.7%) patients, respectively. Since EGFR is expressed not only in cancer cells but also in normal tissues such as the skin, attention must be paid to the risk of photosensitivity. Use of shadowless surgical lights should be avoided during photoimmunotherapy procedures according to the distributor’s manual, and only a headlight, which provide lower light intensity than shadowless surgical lights, can be used during the treatment, although the safety of headlight use during photoimmunotherapy has not yet been rigorously established. At this point, a dilemma emerges: although surgical intervention is often necessary to avoid complications, using only a headlight may not provide sufficient visualization of the surgical field. Therefore, development of a device that would allow clear visualization of the surgical field without activating the drug is desirable.

ORBEYE (Olympus) is a 4K 3D exoscope that provides a magnified 3D surgical field through polarized glasses. The usefulness of ORBEYE has been reported for the treatment of lesions in the head and neck regions including the thyroid, parathyroids, and salivary glands [6–8]. Therefore, if ORBEYE can be used without attendant excitation of the APC, it could prove useful as surgical support device during photoimmunotherapy. Since IR700 has a sharp excitation peak around a wavelength of 690 nm, surgical light with blockade of this excitation peak can be beneficial for surgical procedures performed during photoimmunotherapy. The aim of this study was to design an excitation-light blocking filter tailored to ORBEYE in order to ensure safe surgery during photoimmunotherapy.

## Materials and methods

### Development of the filter tailored to ORBEYE

The LED light source of ORBEYE contains a wavelength component of 690 nm, which is the excitation wavelength of APC. If the excitation wavelength of APC were to be blocked using a general filter, a wide range of wavelengths will be blocked, causing color changes in the images obtained by ORBEYE and resulting in unnatural images, and it would be difficult to obtain clear visualization of the surgical field. To address this issue, a light source filter using a dielectric multilayer filter (Edmund Optics OD4.0 Short Pass Filter, Cutoff Frequence 650 nm) that selectively blocks light near the APC excitation wavelength was developed. This reduces color changes in images and enables natural images to be obtained to allow recalibration of ORBEYE’s white balance.

### Color assessment

The color changes occurring with the developed light source filter were evaluated in images obtained with ORBEYE. The color of the images was evaluated under various conditions; with and without the filter, and with/without re-adjustment of the white balance. The color was also assessed with a color checker (Calibrite LLC). The luminance of each of the 24 colors was measured using the ImageJ software (http://rsb.info.nih.gov/ij/), [9], and the corresponding RGB values were obtained. The absolute differences between the values with and without the filter for each color channel were compared.

### In vitro assessment of cytotoxicity

IR700 was obtained from Rakuten Medical, and cetuximab was purchased from Merck Biopharma. The APC was prepared as previously reported [10]. The cetuximab-IR700 conjugate is hereafter abbreviated as cet-IR700. EGFR expressing HSC-3 (human oral cancer) cells were purchased from the JCRB cell bank [11]. The cells were cultured in RPMI 1640 (FUJIFILM Wako) supplemented with 10% fetal bovine serum and 1% penicillin/streptomycin in a humidified incubator at 37 °C in a 95% air and 5% CO_2_ atmosphere. Fifty thousand cells were seeded on to each well of a 24-well plate. On the following day, the cells were incubated with 10 μg/mL of cet-IR700 for 30 min in a CO_2_ incubator at 37 °C. Then, after the cells were washed with PBS, phenol-red-free medium was added to each well. The cells were then illuminated with ORBEYE from a distance of 50 cm for 15 min, with or without the filter. Cell viability was assessed by the CCK8 assay (FUJIFILM Wako) 2 h after the illumination.

### Clinical evaluation of the filter

The filter was used with ORBEYE for the surgical procedures performed during photoimmunotherapy. It was set to the head of the ORBEYE and white-balanced. The surgeons performed their surgical interventions by watching a 4K monitor through polarized glasses or by direct observation. The ORBEYE settings were used at their default values, and the camera head was positioned optimally for each procedure. The skin area exposed to the ORBEYE light was carefully checked after the treatment. This study was conducted with the approval of the Institute of Science Tokyo Hospital Evaluation Committee for Unapproved New Drugs and Medical Devices (2024-004). Written informed consent was obtained from each of the participating patients.

### Statistical analysis

Data are expressed as the means ± SEM. Statistical analysis was conducted using GraphPad Prism 10 (GraphPad Software). Cell viability was assessed by one-way ANOVA and Dunnett’s test. Differences in luminance were assessed by two-way ANOVA with Sidak’s test. *P* < 0.05 was considered as being indicative of significance.

## Results

### Development of the filter tailored to the ORBEYE exoscope

**Fig. 1:**
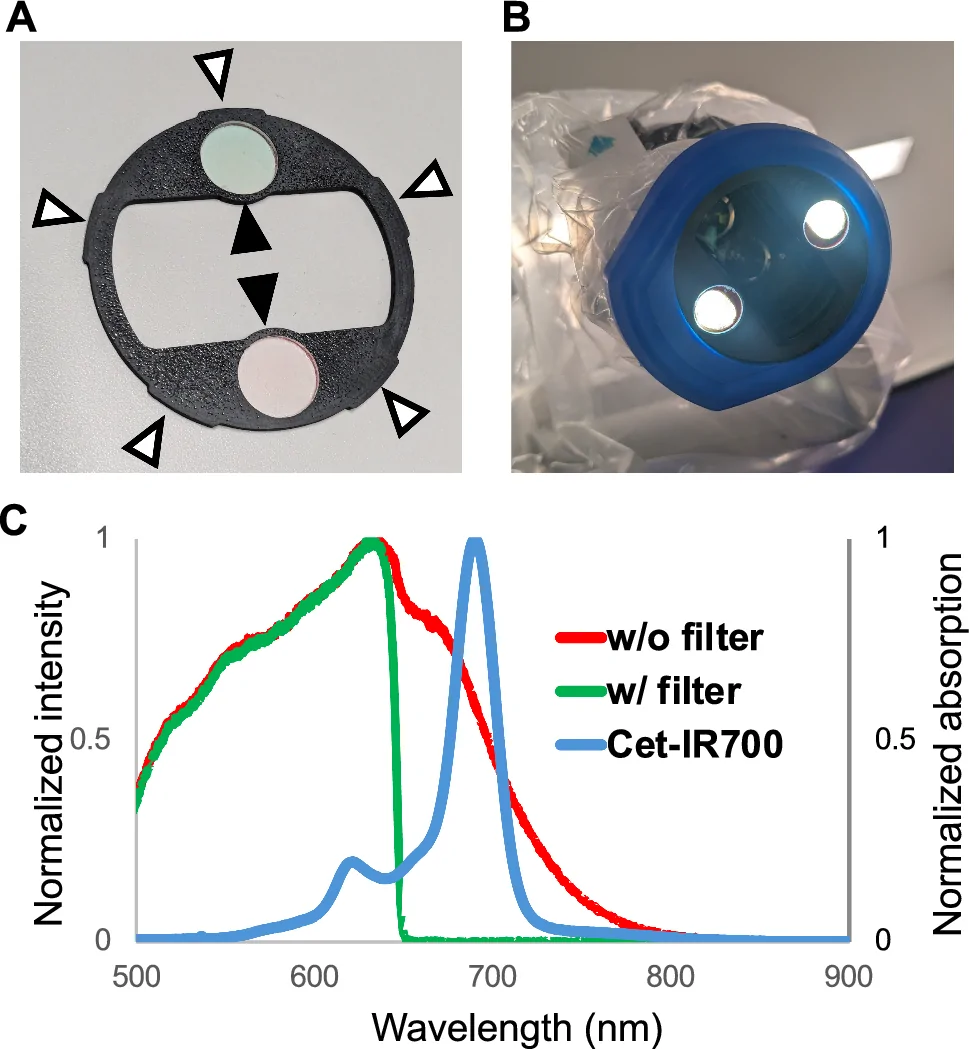
Excitation-light blocking filter tailored to ORBEYE. **A** Two short-pass filters (black arrowheads) were mounted onto the black mounting plate. Five small protrusions (white arrowheads) were designed around the perimeter of the black mounting plate to fit into the indentations on the inner side of ORBEYE's sterile cover.** B** When attached to the ORBEYE, the two short-pass filters were positioned directly above ORBEYE's light source. **C** Normalized intensity of the ORBEYE light with (w/) and without (w/o) the filter and normalized absorption of cetuximab sarotalocan sodium (cet-IR700). Using the filter for ORBEYE light effectively eliminated the cet-IR700 excitation light from the observation illumination

The filter was manufactured using the dielectric multilayer film deposition technology. Two filters were placed on the mounting plate to cover two of ORBEYE’s light sources and to block the light beyond 650 nm from ORBEYE (Fig. 1A). In order to fix the filter assembly to notches placed in the inner side of the sterile drape for ORBEYE, 5 small projections were designed. The filter assembly was confirmed as being fixed within the sterile drape for ORBEYE (Fig. 1B). The light spectrum from ORBEYE was measured with and without the filter, and we found that the filter successfully blocked light beyond 650 nm wavelength from ORBEYE. The blocked wavelengths were shown to block the excitation light of cet-IR700 (Fig. 1C).

### Color assessment

**Fig. 2:**
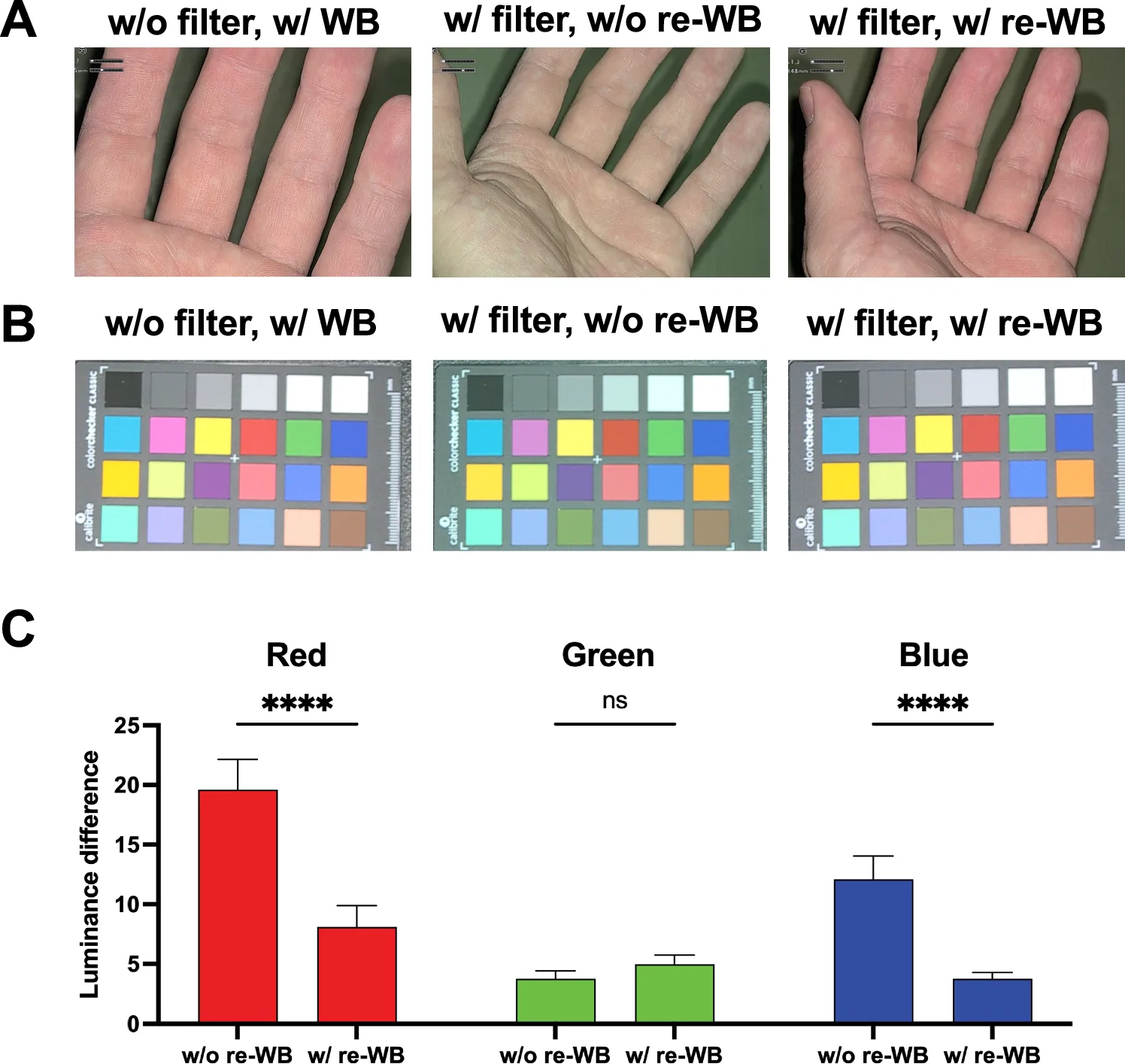
Color assessment. **A** The hand was observed under three conditions: normal observation without a filter, filter set without (w/o) re-adjustment of the white balance (WB), filter set with (w/) re-adjustment of the white balance. **B** Observation using the color checker was performed under the same conditions (Fig. 2A). **C** The luminance of each of the 24 colors was measured using ImageJ, and the corresponding RGB values were extracted. The RGB values obtained without the filter were used as the reference values, and the absolute differences from these reference values were calculated for each color channel under each condition (n = 24; Two-way ANOVA with Sidak’s test; ****, < 0.0001; ns, not significant)

The color visibility was assessed. First, the hand was observed under ORBEYE light that was white-balanced, and then it was observed under ORBEYE light used with the filter without re-adjustment of the white balance. The hand now appeared slightly bluish (Fig. 2A). Thirdly, the hand was observed again under ORBEYE light used with the filter after re-adjustment of the white balance, which showed no noticeable color distortion. The color was assessed with the color checker in the same way. A 24-color panel was observed under ORBEYE light (Fig. 2B, sFig. 1). With recalibration of the white balance, the luminance values for red and blue became closer to those obtained without the filter. In contrast, the difference in luminance for green was already small even without the recalibration (Fig. 2C).

### In vitro assessment of cytotoxicity

**Fig. 3:**
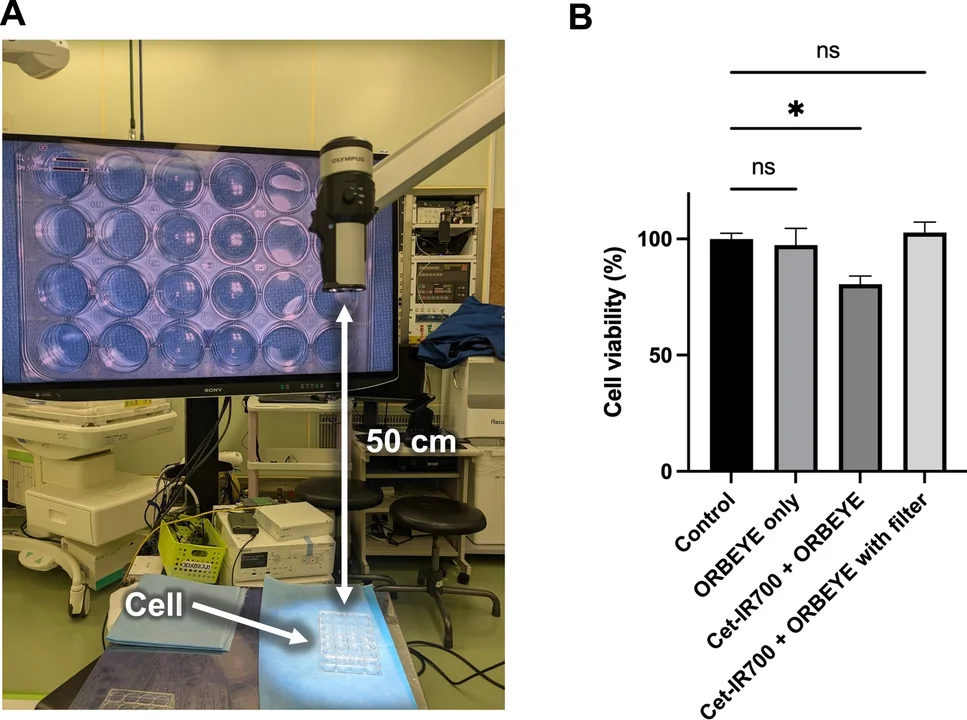
In vitro assessment of cytotoxicity. **A** The cells were illuminated with ORBEYE light from a distance of 50 cm for 15 min. **B** The cell viability was assessed by the CCK8 assay (n = 4; one-way ANOVA with Dunnett’s test; *, *p* < 0.05; ns, not significant; vs. control)

In order to assess if use of the filter could reduce the potential cytotoxicity during photoimmunotherapy, the cell viability was evaluated by the CCK8 assay (Fig. 3). Light exposure from ORBEYE without cet-IR700 was associated with no cytotoxicity as compared with the control, while exposure to both cet-IR700 and light from ORBEYE without the filter was associated with significantly reduced viability of the HSC-3 cells. On the other hand, no decrease in the cell viability, as compared with the control, was observed when the cells were exposed to cet-IR700 and light from ORBEYE with the filter. These results suggest that the cytotoxicity exerted by cet-IR700 plus ORBEYE light was abrogated by use of the filter.

### Clinical evaluation of the filter

**Fig. 4:**
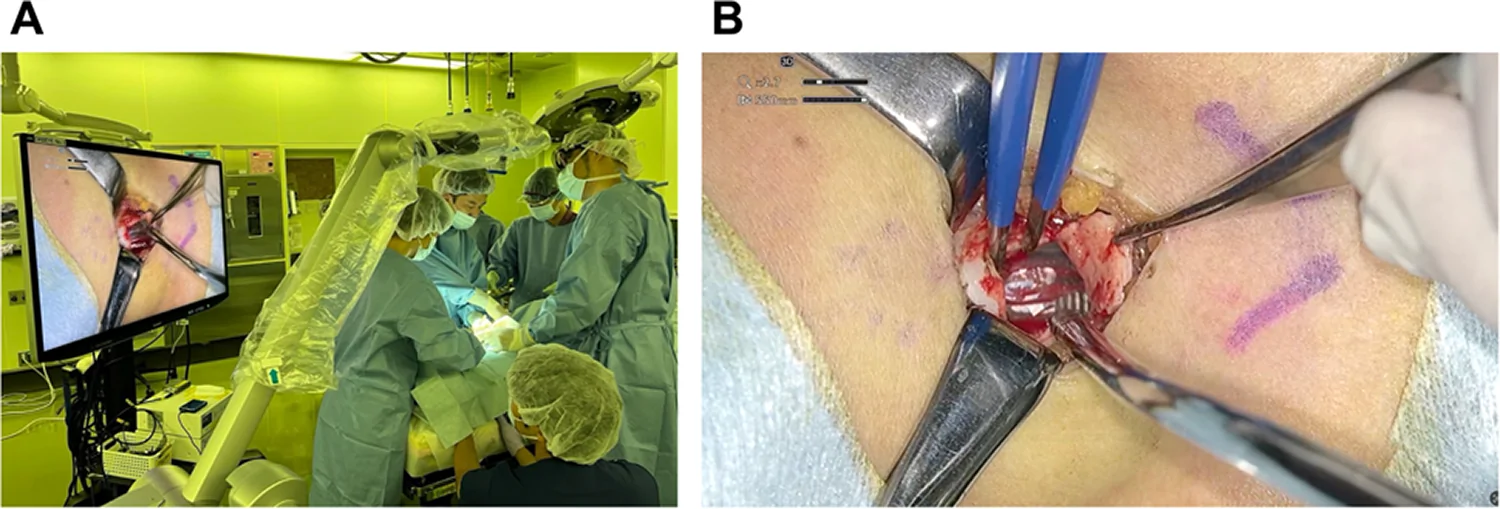
Clinical usage of the filter. **A** Typical view during an operation under ORBEYE using a filter. **B** No perceptible alteration in color tone was detected

We used the ORBEYE light with the filter for six patients who underwent photoimmunotherapy. The characteristics of the patients are shown in Table 1. The median age of the six patients was 67.5 [range: 55–81] years; two of the patients were female and four were male. The primary tumor site was the oropharynx in five cases and the hypopharynx in one case. In three cases, the treatment target was the base of the tongue; in one each of the remaining cases, it was the postcricoid area, the lateral wall of the oropharynx, and a lymph node. The histological type was squamous cell carcinoma (SCC) in all cases. In three patients, the ORBEYE was used at two anatomical sites each, for a total of nine sites. Tracheostomy was performed in 5 out of the 6 cases. Arterial ligation was performed in 3 cases, while lymph node dissection and tumor debulking were each performed in 1 case. The procedure duration ranged from 9 to 53 min. By digitally correcting the bluish discoloration caused by the filter, the system provides a natural surgical view comparable to that of standard surgical procedures (sMovie 1). The typical device setting is shown in Fig. 4A. The operator watched a monitor or performed direct observation under the ORBEYE light. All the procedures were completed without any problems, and no skin problems, including erythema or rash, were observed at the sites where ORBEYE was used during at least one week of daily postoperative assessments.

**Table 1:** Characteristics of the patients

Case	Age	Sex	Primary site	Target of PIT	Histology	Procedure with ORBEYE	Duration (min)
1	55	F	Hypopharynx	Postcricoid area	SCC	Tracheostomy	9
2	56	M	Oropharynx	Lateral wall of oropharynx	SCC	Tracheostomy	13
3	81	M	Oropharynx	Base of tongue	SCC	Tracheostomy; Arterial ligation	14; 47
4	67	M	Oropharynx	Base of tongue	SCC	Tracheostomy; Arterial ligation + lymph node extraction	13; 52
5	70	M	Oropharynx	Base of tongue	SCC	Tracheostomy, Arterial ligation	17; 53
6	68	F	Oropharynx	Lymph node	SCC	Tumor debulking	21

F, Female; M, Male; SCC, Squamous cell carcinoma

## Discussion

We developed a filter designed for the ORBEYE exoscope that blocks the excitation light for photoimmunotherapy. The peak absorbance wavelength of IR700 is 690 nm and the tails are narrow. Blocking light with wavelengths above 650 nm is thought to reduce cytotoxicity by preventing IR700 photoactivation caused by the observation light, thereby enabling safe surgery during photoimmunotherapy. We set small two short-pass filters, almost the same size as the light illuminators of ORBEYE, to a mounting plate. Minimizing the filter size allowed for cost reduction. The mounting plate featured a hollow center to avoid covering the camera region, which helped to minimize its effect on color rendering. Additionally, by enabling placement within the sterile drape, the device could be operated while preserving a sterile environment. Color tone was evaluated using human skin and a color checker. The results indicated that, although the image appeared bluish when viewed through the filters without adjustment, recalibrating the white balance after applying the filters enabled reproduction of a natural and consistent color tone. Cytotoxicity assessment demonstrated that 15 min of ORBEYE light exposure in the presence of cet-IR700 induced statistically significant cellular damage in the HSC-3 cells. This effect, however, was abrogated by application of the filter to the light. The system was applied in actual patients and was successfully utilized across various surgical procedures without any complications. No cases of postoperative skin damage were observed. These findings suggest that we successfully developed a device that allows for surgical procedures to be performed safely under ORBEYE light during photoimmunotherapy without inducing cytotoxicity.

In this study, we used the device only for minor procedures such as tracheostomy and vascular ligation. However, since its safety has been confirmed, we believe it could also be applied to more extensive procedures in the future. For instance, in cases requiring both photoimmunotherapy and neck dissection, previously, we performed neck dissection first and then undertook photoimmunotherapy on a separate day [12]. With the use of the present device, it may become possible to perform both procedures on the same day. Moreover, in cases of lymph node metastasis with extranodal extension, it may be feasible to surgically remove the tumor as much as possible through neck dissection and subsequently apply intraoperative photoimmunotherapy to the dissected area, which may contribute to better therapeutic outcomes. In some cases, it is necessary to sufficiently expose the surgical field for effective light delivery during photoimmunotherapy. The device is expected to enable such procedures to be performed safely and easily.

IR700 is a fluorescent dye whose emission, upon excitation by therapeutic laser light, can be detected using infrared fluorescence imaging systems [13]. Moreover, monitoring of fluorescence attenuation during treatment provides valuable feedback for optimizing the delivered therapeutic light dose [14–16]. The ORBEYE system is equipped with an infrared imaging mode, enabling real-time visualization of IR700 fluorescence during photoimmunotherapy (data not shown). Consequently, the ORBEYE exoscope serves not only as a surgical support tool during photoimmunotherapy, but also as an effective fluorescence imaging device, underscoring its suitability and synergy with this treatment modality.

The limitations of this study should be acknowledged. In the present study, the device was used only for minor procedures that could be completed within 60 min. Further investigation is needed to determine whether complications may arise with the use of ORBEYE light with the filter during longer surgical procedures such as neck dissection. However, since the cytotoxic effects were successfully mitigated at the cellular level, it is likely that such procedures can also be performed safely.

In conclusion, we developed a filter for the ORBEYE system that is designed to selectively block the excitation light for photoimmunotherapy. We validated its color fidelity and safety through in vitro cellular assays and clinical use in patients, demonstrating its usefulness. This device is expected to expand the therapeutic applications of photoimmunotherapy in the future, particularly by enabling treatment strategies that combine surgery and photoimmunotherapy.

## Supplementary Information

Below is the link to the electronic supplementary material.

Supplementary file1 (MP4 125627 KB)

Supplementary file2 (PPTX 130 KB)

## Data Availability

The data supporting the findings of this study are not publicly available due to the inclusion of personal information; however, data that do not contain personal information are available from the corresponding author upon reasonable request.
